# Long‐Term Fluctuations in Cetacean Occurrence Derived From Whale‐Watching Platforms Off Southwest Gran Canaria: Evaluating Their Potential as an Ecological Indicator

**DOI:** 10.1002/ece3.74232

**Published:** 2026-09-01

**Authors:** Inma Herrera, Antonio G. Ramos, Ángel Rodríguez‐Santana, Ricardo Haroun

**Affiliations:** ^1^ Research Group in Biodiversity & Conservation (BIOCON) University Institute ECOAQUA, University of Las Palmas de Gran Canaria (ULPGC), Scientific & Technological Marine Park Telde Spain; ^2^ Oceanografía Física y Geofísica Aplicada (OFYGA) University Institute ECOAQUA, University of Las Palmas de Gran Canaria (ULPGC) Tafira Spain

**Keywords:** cetaceans, chlorophyll‐*a*, Gran Canaria, Macaronesia, mother‐calf groups, relative occurrence, Special Areas of Conservation, SPUE, whale‐watching platforms

## Abstract

Long‐term variability in cetacean detection patterns in oceanic archipelagos remains poorly documented, limiting our understanding of temporal dynamics and their relevance for marine conservation and management. Here, we analysed an 18‐year dataset (2004–2025) comprising 9275 cetacean detections collected from whale‐watching platforms off south‐western Gran Canaria (Canary Islands, NE Atlantic). Twenty cetacean species were detected, with odontocetes dominating the records, marked seasonal variability and recurrent detection of mother–calf groups of *Stenella frontalis* and *Tursiops truncatus* in coastal waters. Species‐specific models revealed contrasting temporal trajectories among taxa, while direct relationships between monthly detections and sea surface temperature or chlorophyll‐*a* concentration were limited after accounting for temporal variability and sampling effort. Effort‐standardised sightings per unit effort (SPUE) showed substantial temporal variability, including increased detection rates during 2013–2019, a marked reduction during the COVID‐19 period, and variable detection rates thereafter, reflecting the combined influence of sampling disruption and natural variability in cetacean detection patterns. Spatial analyses showed overlap between areas of recurrent cetacean detections and existing Special Areas of Conservation (SACs), while additional areas of frequent detections were identified outside current protected boundaries. Our results demonstrate the value of long‐term whale‐watching datasets as complementary sources of information for assessing cetacean temporal and spatial variability. Although SPUE provides a cost‐effective index of effort‐standardised detection patterns, further validation with independent monitoring approaches is required before it can be incorporated as a robust ecological indicator.

## Graphical abstract

Using an 18‐year dataset of whale‐watching observations from Gran Canaria (Canary Islands), we analysed long‐term temporal dynamics of cetacean communities in the subtropical Atlantic. Standardised sightings per unit effort (SPUE) revealed strong seasonal patterns, multi‐year fluctuations and species‐specific responses to environmental variability. These results highlight the value of whale‐watching platforms as cost‐effective tools for long‐term biodiversity monitoring and marine conservation.

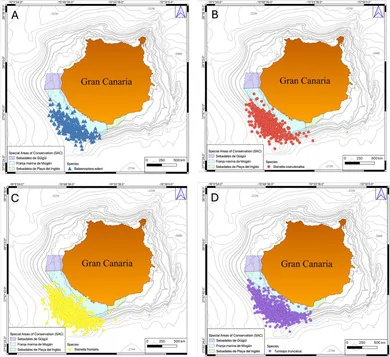

## 1 Introduction

Cetaceans play a fundamental ecological role in marine ecosystems and are increasingly recognised as sensitive indicators of environmental change, trophic dynamics and anthropogenic pressures (Piroddi et al. 2022). Long‐term datasets are essential for detecting population trends, supporting the development of biodiversity indicators, and informing ecosystem‐based management. However, such time series remain scarce in oceanic archipelagos, where maintaining continuous scientific monitoring is challenging due to logistical and financial constraints (González García et al. 2023; Lacetera et al. 2023). Consequently, despite the growing need for biodiversity indicators, particularly in the context of the EU Marine Strategy Framework Directive (MSFD), long‐term ecological datasets for oceanic ecosystems remain limited, and only a few have been incorporated into management and policy frameworks.

The eastern subtropical Atlantic, particularly the Canary Islands, represents one of the most important cetacean biodiversity hotspots in the Northern Hemisphere, with at least 30 documented species (Herrera et al. 2021; Martín et al. 2024). The oceanography of the region is strongly influenced by the Canary Current, the subtropical gyre, and episodic coastal upwelling off northwest Africa. In addition, the steep island bathymetry and the formation of mesoscale eddies around the archipelago contribute to local productivity patterns and water‐mass retention, potentially creating favourable conditions for a diverse assemblage of marine predators (Arístegui et al. 2009; Sangrà et al. 2007). This dynamic environment supports a diverse community of resident, migratory and seasonal cetacean species, highlighting the importance of long‐term monitoring for understanding ecological dynamics across spatial and temporal scales.

Despite its ecological importance, the waters off Gran Canaria have received considerably less research attention than those surrounding neighbouring islands such as Tenerife and La Gomera. This geographical bias has limited our understanding of temporal variability, habitat use and ecological dynamics in this sector of the archipelago (Canarias Coservation, n.d.). As a result, our ability to detect long‐term changes and provide robust scientific information to support conservation and management remains limited, particularly in an area increasingly exposed to anthropogenic pressures such as intense maritime traffic and the continued growth of whale‐watching activities (Bejder et al. 2006; Fais et al. 2016).

Platforms of opportunity (PoO), such as whale‐watching vessels, provide a cost‐effective means of generating long‐term datasets on cetacean occurrence patterns when systematic recording protocols and standardised sampling procedures are applied (Alves et al. 2013, 2018; Arranz et al. 2023). In the Macaronesian region, these platforms have contributed substantially to understanding cetacean seasonality, areas of recurrent use, habitat use and species–environment relationships (Alves et al. 2018; Thompson et al. 2015). Because cetacean occurrence is influenced by multiple environmental drivers operating across different spatial and temporal scales, incorporating variables describing oceanographic conditions and primary productivity may improve our understanding of species‐specific occurrence patterns. However, the strength of these relationships may vary among species depending on ecological traits, behaviour, distribution and observation processes.

The waters southwest of Gran Canaria encompass three Special Areas of Conservation (SACs) and therefore represent an important area for investigating long‐term patterns of cetacean occurrence and their relationship with protected areas. In this context, long‐term monitoring datasets can provide valuable information to support conservation planning and adaptive management of subtropical marine ecosystems.

In the present study, we analysed an 18‐year dataset (2004–2025) comprising 9275 standardised cetacean sightings collected from whale‐watching platforms off southwest Gran Canaria. This study aimed to: (Almunia et al. 2021) describe the long‐term species composition of cetaceans in the study area; (Alves et al. 2013) characterise the spatial and temporal patterns of cetacean occurrence; (Alves et al. 2018) quantify seasonal and interannual variability using standardised sightings per unit effort (SPUE); (Aguilar de Soto et al. 2020) evaluate associations between cetacean occurrence patterns and environmental variability, including sea surface temperature and chlorophyll‐*a* concentration, using generalised additive modelling approaches; and (Arranz et al. 2023) assess the potential contribution of SPUE derived from whale‐watching platforms as a complementary metric for long‐term cetacean monitoring and conservation assessments. By combining long‐term occurrence records with environmental and spatial analyses, we evaluate the capacity of whale‐watching datasets to characterise temporal variability in cetacean occurrence and discuss their applicability within broader marine monitoring frameworks.

## 2 Materials and Methods

### 2.1 Study Area and Data Collection

This study was conducted in the waters southwest of Gran Canaria (Canary Islands, eastern Atlantic Ocean), a region characterised by complex bathymetry, narrow continental shelf areas and oceanographic conditions influenced by the Canary Current and subtropical circulation patterns. The study area includes coastal and oceanic habitats extending from the island shelf to deeper offshore waters, encompassing areas frequently used by whale‐watching operations.

Cetacean sighting data were collected between 2004 and 2025 by the whale‐watching company Spirit of the Sea, operating from the southwestern coast of Gran Canaria. The dataset represents a long‐term platform‐of‐opportunity (PoO) monitoring programme, in which commercial whale‐watching trips were used as a source of standardised cetacean observations. Crew members involved in data collection had extensive experience in cetacean identification and marine observations.

During the study period, 9275 cetacean detections were recorded, representing 20 species. Surveys were conducted following the code of conduct established by the Government of the Canary Islands (Decree 178/2000; Royal Decree 1727/2007). Whale‐watching surveys followed a predefined navigation route within the southwestern waters of Gran Canaria, with comparable survey duration among trips throughout the study period. Although the surveyed route remained consistent, the precise locations of cetacean detections varied according to the distribution of animals encountered during each survey.

For each detection event, the following information was recorded: date, time, geographic coordinates, species identification, group size, presence of calves and distance from the coast, among other variables. Species were identified to the lowest possible taxonomic level using standard field guides and taxonomic references (e.g., Carwardine 1995). Although group size and other biological information were available, the analyses presented here focused on detection frequency standardised by sampling effort rather than direct estimates of abundance.

**FIGURE 1:**
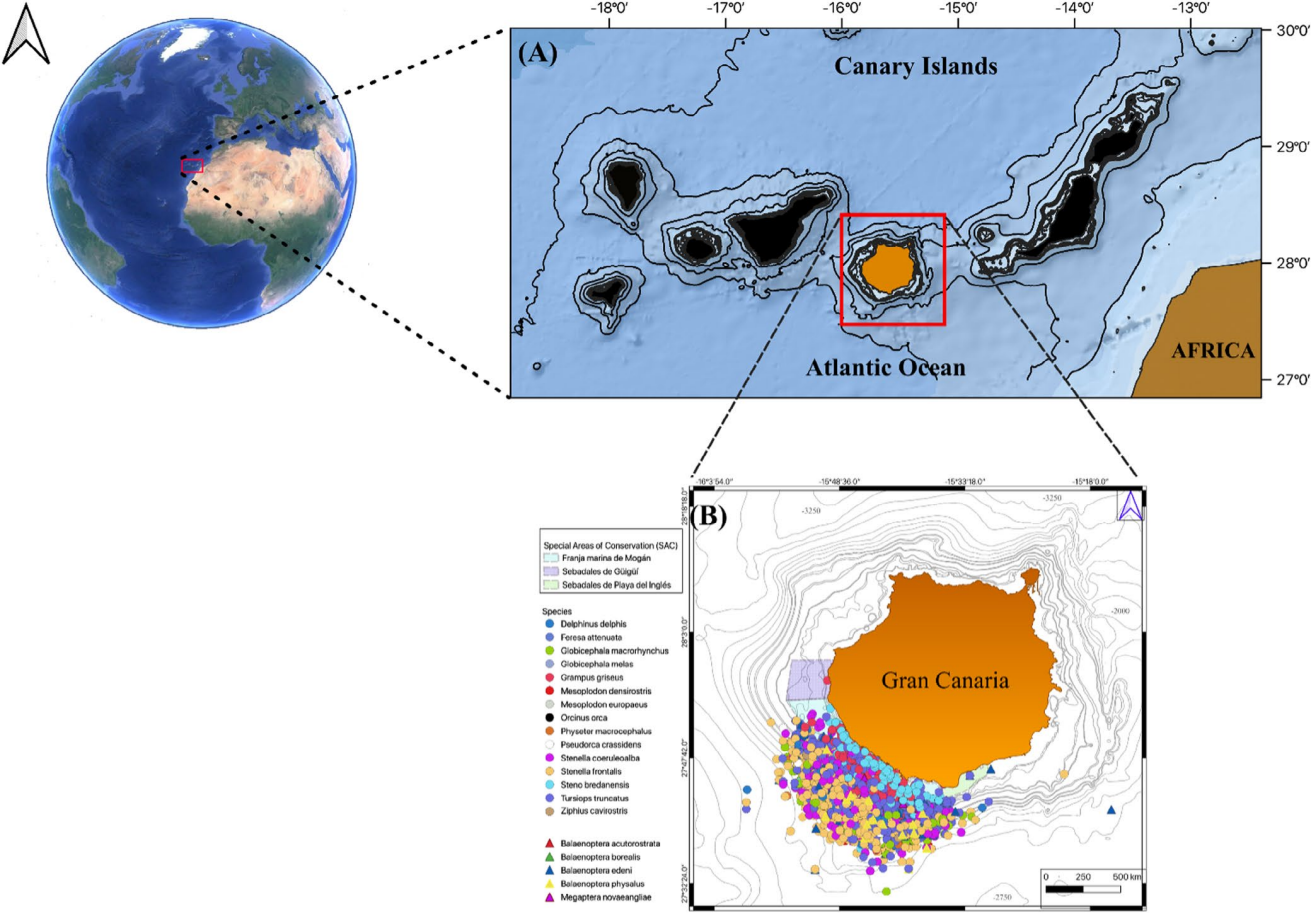
(A) Location of the Canary Islands in the eastern Atlantic Ocean. (B) Spatial distribution of cetacean detections recorded by the whale‐watching platform between 2004 and 2025 in the southwestern waters of Gran Canaria. Coloured points represent recorded detection events and polygons indicate Special Areas of Conservation (SACs).

The study area overlaps with three Special Areas of Conservation (SACs; Zona Especial de Conservación, ZEC): Franja Marina de Mogán, Sebadales de Playa del Inglés and Sebadales de Güigüi. The latter has been proposed as part of a future marine–terrestrial national park due to its high biodiversity value and ecological importance (Figure 1).

### 2.2 Environmental and Biological Variables

To complement cetacean observations, environmental variables were obtained from Copernicus Marine Environment Monitoring Service (CMEMS 2025) products. Daily environmental fields were extracted for the study period (2004–November 2025) and matched to each cetacean sighting according to the date and geographic coordinates of each observation.

Sea surface temperature (SST, °C) was obtained from the CMEMS Global Ocean Physics Analysis and Forecast product (GLOBAL_ANALYSISFORECAST_PHY_001_024), which provides daily estimates of surface temperature at a spatial resolution of 0.083° × 0.083°. SST was selected as an indicator of oceanographic thermal variability because temperature gradients influence marine ecosystem processes, including prey distribution and habitat suitability for upper trophic‐level predators (Correia et al. 2021; Pérez‐Jorge et al. 2020).

Chlorophyll‐*a* concentration (CHL, mg m^−3^) was obtained from the CMEMS Atlantic‐Iberian Biscay Irish Ocean Biogeochemical Analysis and Forecast product (IBI_ANALYSISFORECAST_BGC_005_004), with a spatial resolution of 0.028° × 0.028° (Gutknecht et al. 2019). CHL was used as a proxy of phytoplankton biomass and primary productivity, representing a bottom‐up indicator of potential changes in marine food‐web dynamics that may indirectly affect cetacean prey availability.

For each sighting event, SST and CHL values were extracted from the corresponding CMEMS grid cell. Environmental variables were subsequently aggregated at a monthly scale to match the temporal resolution used in the occurrence models. Chlorophyll‐*a* values were log‐transformed prior to modelling to reduce skewness and account for the highly variable distribution of productivity values.

### 2.3 Data Processing and Statistical Analyses

Sighting records were processed to characterise species composition, temporal variability and spatial distribution patterns. To account for differences in survey effort among months, sightings per unit effort (SPUE) were calculated as the number of species‐specific sightings divided by the number of survey days within each month. SPUE was used as a descriptive index of relative detection rates, allowing comparisons of species occurrence patterns under heterogeneous sampling effort. Species were further classified into two major taxonomic groups (Odontoceti and Mysticeti).

To assess temporal and environmental drivers of cetacean detection patterns while accounting for variation in sampling effort, a negative binomial Generalised Additive Model (GAM; Wood 2017) was fitted to monthly sighting counts of the four focal species. Although cetacean observations were available from 2004 onwards, GAM analyses were restricted to the period 2011–2025 because continuous monthly survey effort data required for modelling were only available during this period. The response variable consisted of monthly species‐specific sighting counts, with the logarithm of monthly survey effort included as an offset term to account for differences in sampling intensity among months.

The GAM included species identity as a parametric factor to account for differences in baseline sighting rates among taxa, a smooth temporal component representing nonlinear changes throughout the monitoring period, cyclic seasonal variation (month) and smooth functions of environmental predictors including sea surface temperature (SST) and chlorophyll‐*a* concentration (CHL). Temporal variation was represented by a continuous index calculated as the number of months elapsed since January 2011 (*Time_index*), with each month corresponding to one temporal step in the model. This index represents temporal progression during the monitoring period and does not refer to the time of individual sightings.

Smooth terms were fitted using penalised regression splines under restricted maximum likelihood (REML). Model family selection was based on dispersion diagnostics comparing Poisson and negative binomial distributions. The negative binomial family was selected because sighting counts showed overdispersion relative to the Poisson assumption.

Model performance was evaluated using approximate significance tests for smooth terms, Wald tests for parametric coefficients, percentage deviance explained, adjusted *R* ^2^ and diagnostic evaluation of residual behaviour and basis dimension adequacy. Separate negative binomial GAMs were subsequently fitted for each focal species ( *Balaenoptera edeni* , *Stenella coeruleoalba* , *Stenella frontalis* and *Tursiops truncatus* ) to characterise species‐specific temporal patterns.

All statistical analyses were performed in R version 4.3.0 (R Core Team 2023) using the packages *mgcv* (Wood 2017), *nlme* (Pinheiro et al. 2023) and *tidyverse*. Spatial distribution maps were produced using QGIS version 3.16 (Hannover; QGIS Development Team 2021). The four most frequently detected species were mapped individually together with Special Areas of Conservation (SACs) to evaluate spatial overlap between cetacean distribution patterns and existing protected areas.

## 3 Results

### 3.1 Species Composition and Sightings

Between 2004 and 2025, whale‐watching surveys were conducted with variable annual sampling effort (Table S1). No survey data were available for 2005, 2006, 2009 and 2010, whereas continuous monitoring was achieved from 2011 onwards, with annual effort ranging from 63 to 346 survey days. Across the study period, a total of 4260 survey days resulted in 9275 cetacean sightings representing 20 species: 15 odontocetes and 5 mysticetes (Figure 1; Table S1).

**FIGURE 2:**
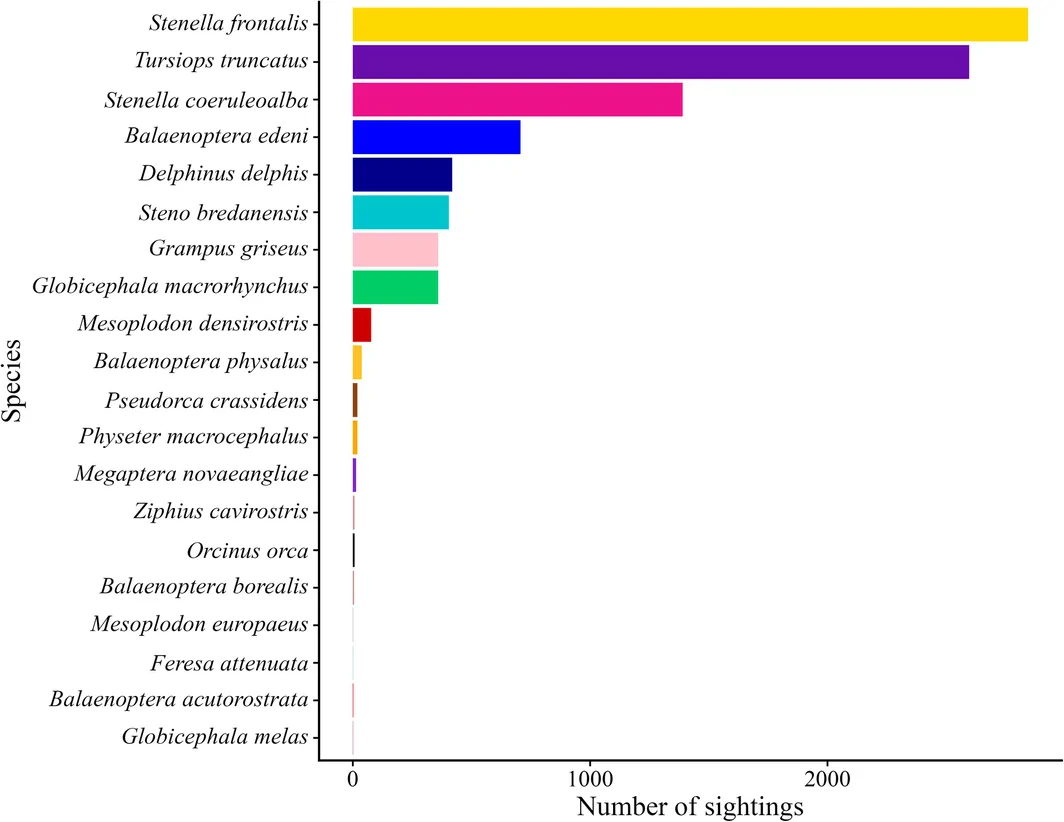
Ranking of cetacean species according to the number of sightings recorded in the southwestern waters of Gran Canaria between 2004 and 2025. Species are ordered from the most to the least frequently observed. The four most common species were selected for subsequent temporal and environmental modelling.

The most frequently detected species were *S. frontalis* (*n* = 2845), *T. truncatus* (*n* = 2597), *S. coeruleoalba* (*n* = 1390) and *B. edeni* (*n* = 707) (Figure 2, Table S1).

### 3.2 Interannual Variation

**FIGURE 3:**
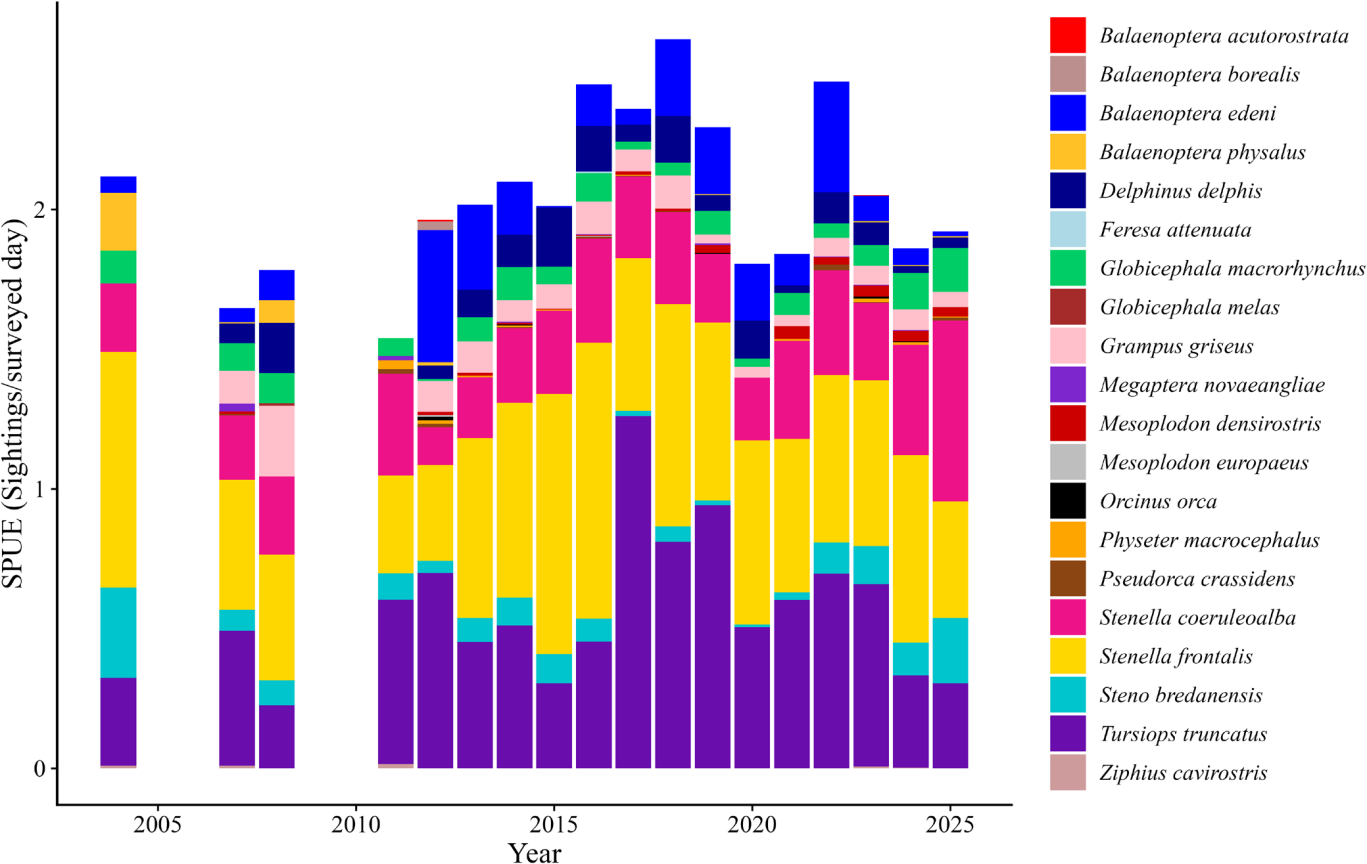
Annual variation in effort‐standardised cetacean detection rates (SPUE; sightings per surveyed day) in the southwestern waters of Gran Canaria between 2004 and 2025. No data were available during 2005, 2006, 2009 and 2010.

The annual time series of effort‐standardised cetacean detection rates (SPUE; sightings per study day) revealed marked interannual variability in cetacean detections in the waters southwest of Gran Canaria between 2004 and 2025 (Figure 3; Table S1). No data were available for 2005, 2006, 2009 and 2010. From 2011 onwards, total SPUE increased progressively, reaching maximum values between 2016 and 2018, followed by subsequent interannual fluctuations. In 2020, a marked decrease in SPUE was observed, coinciding with the disruption of whale‐watching activity associated with the COVID‐19 pandemic and the reduction in sampling effort. Although SPUE accounts for differences in survey effort, variation in spatial coverage, observation conditions and detectability may still influence effort‐standardised detection rates; therefore, this decrease should be interpreted as a reduction in observed detections rather than direct evidence of a decline in cetacean abundance.

Throughout the study period, dolphins accounted for the largest proportion of annual SPUE, particularly *S. frontalis* , *S. coeruleoalba* and *T. truncatus* . *S. frontalis* showed consistently high contributions, with pronounced peaks between 2015 and 2018 (Figure 3).

Large odontocetes, including *Physeter macrocephalus* and *Ziphius cavirostris* , also contributed regularly, with higher values mainly between 2016 and 2019 and again after 2022. Baleen whales (*Balaenoptera* spp.) were recorded sporadically, with isolated annual peaks driven largely by *B. edeni* and, occasionally, *B. physalus* . Overall, odontocetes dominated the annual records, with 15 species compared with 5 mysticete species (Figure 3).

### 3.3 Monthly Distribution

**FIGURE 4:**
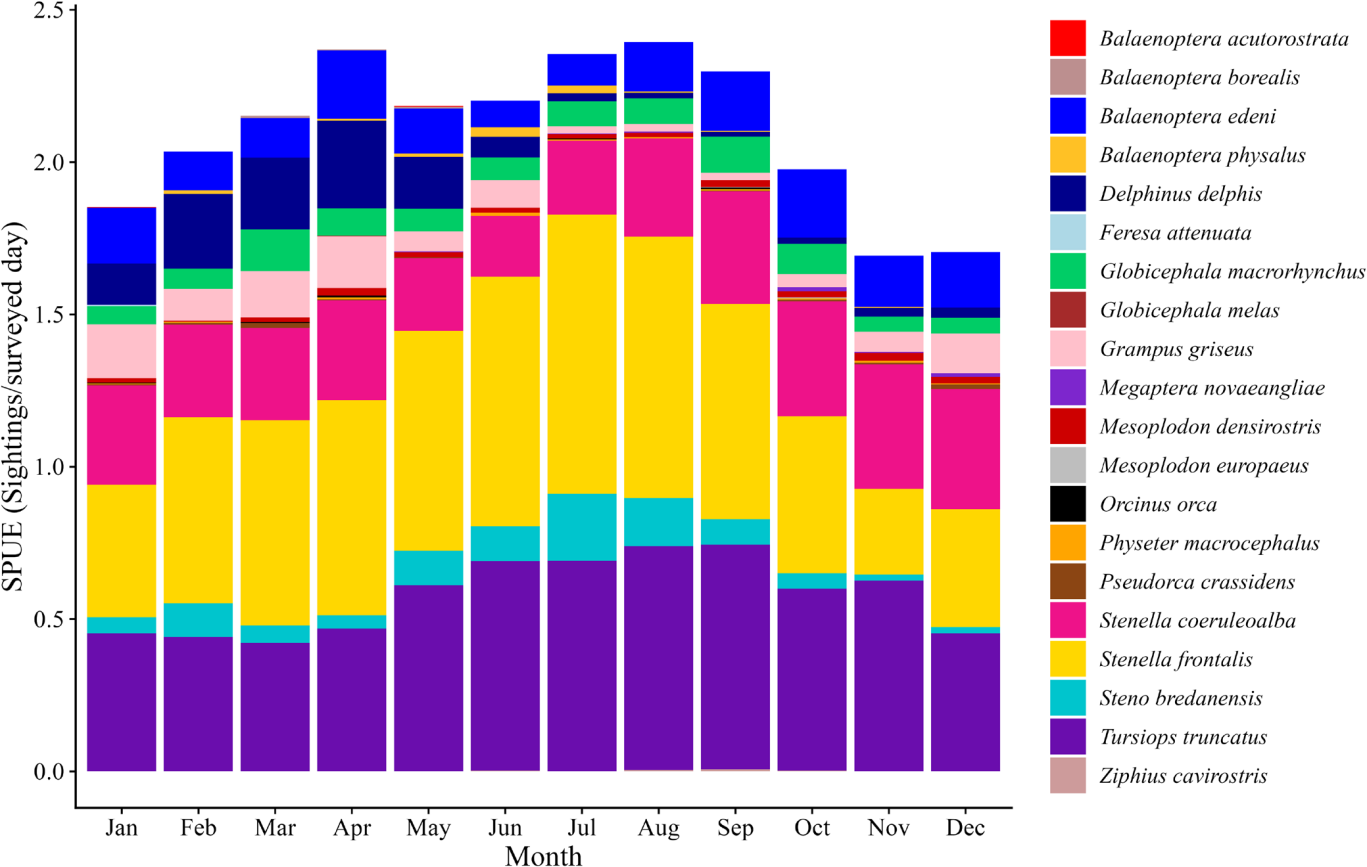
Monthly distribution of effort‐standardised cetacean detection rates (SPUE; sightings per surveyed day) in the southwestern waters of Gran Canaria.

The aggregate effort‐standardised cetacean detection rates (SPUE; sightings per study day) revealed a clear seasonal pattern in cetacean detections in the waters southwest of Gran Canaria (Figure 4). SPUE values were generally lower during winter (January–March), increased progressively during spring, and reached maximum values between late spring and summer (May–September), before declining again during autumn (October–December).

Dolphins contributed the largest proportion of the total monthly SPUE throughout the year, particularly *S. frontalis* , *S. coeruleoalba* and *T. truncatus* . *S. frontalis* showed notably high SPUE values during spring and summer, coinciding with the seasonal peak in overall effort‐standardised detection rates.

Large odontocetes, including *Physeter macrocephalus* and *Ziphius cavirostris* , were detected during most months, with low but persistent SPUE values and slightly higher contributions during summer and early autumn. Baleen whales were mainly represented by *B. edeni* , which showed a pronounced seasonal pattern, with most detections concentrated between late winter and early spring (Figure 4; Table S2).

### 3.4 Species‐Specific Temporal Dynamics

**FIGURE 5:**
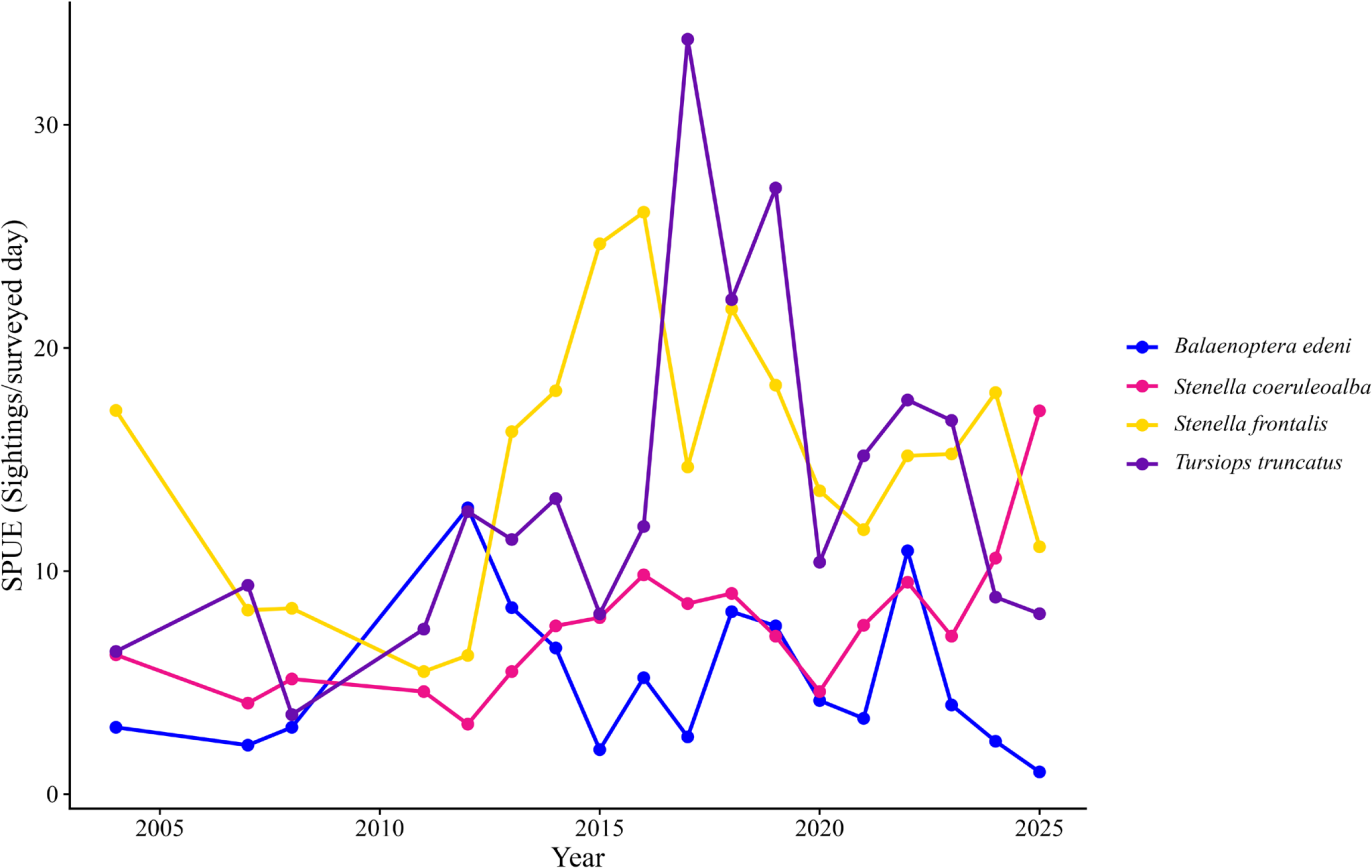
Annual variation in effort‐standardised cetacean detection rates (SPUE; sightings per surveyed day) for the four most frequently recorded cetacean species in the southwestern waters of Gran Canaria between 2004 and 2025. Years without data (2005, 2006, 2009 and 2010) were excluded.

Annual effort‐standardised detection rates (SPUE; sightings per surveyed day) revealed marked interannual variability among the four most frequently recorded species. *T. truncatus* showed increased SPUE values between 2016 and 2019, followed by moderate values in recent years. *S. frontalis* showed its highest SPUE values between 2015 and 2018, after which detection rates declined. In contrast, *S. coeruleoalba* maintained relatively stable SPUE values throughout most of the study period, although with a decline around 2020 and a subsequent partial recovery. The mysticete *B. edeni* showed a more intermittent pattern, with sporadic peaks that did not coincide with those of the dominant odontocetes. This pattern may reflect differences in habitat use, but may also be influenced by species‐specific detectability, including differences in surfacing behaviour, group size and offshore distribution (Figure 5).

### 3.5 Spatial Patterns

The monthly time series revealed species‐specific seasonal patterns and variable degrees of temporal overlap among the main cetacean species. *S. frontalis* tended to show peak detection rates during periods that differed from those of *T. truncatus* , suggesting differences in the seasonal use of coastal habitats. Although the two *S*. species overlapped during several months, their peak detection rates did not always coincide, which may reflect differences in habitat use, prey associations, or ecological strategies.

**FIGURE 6:**
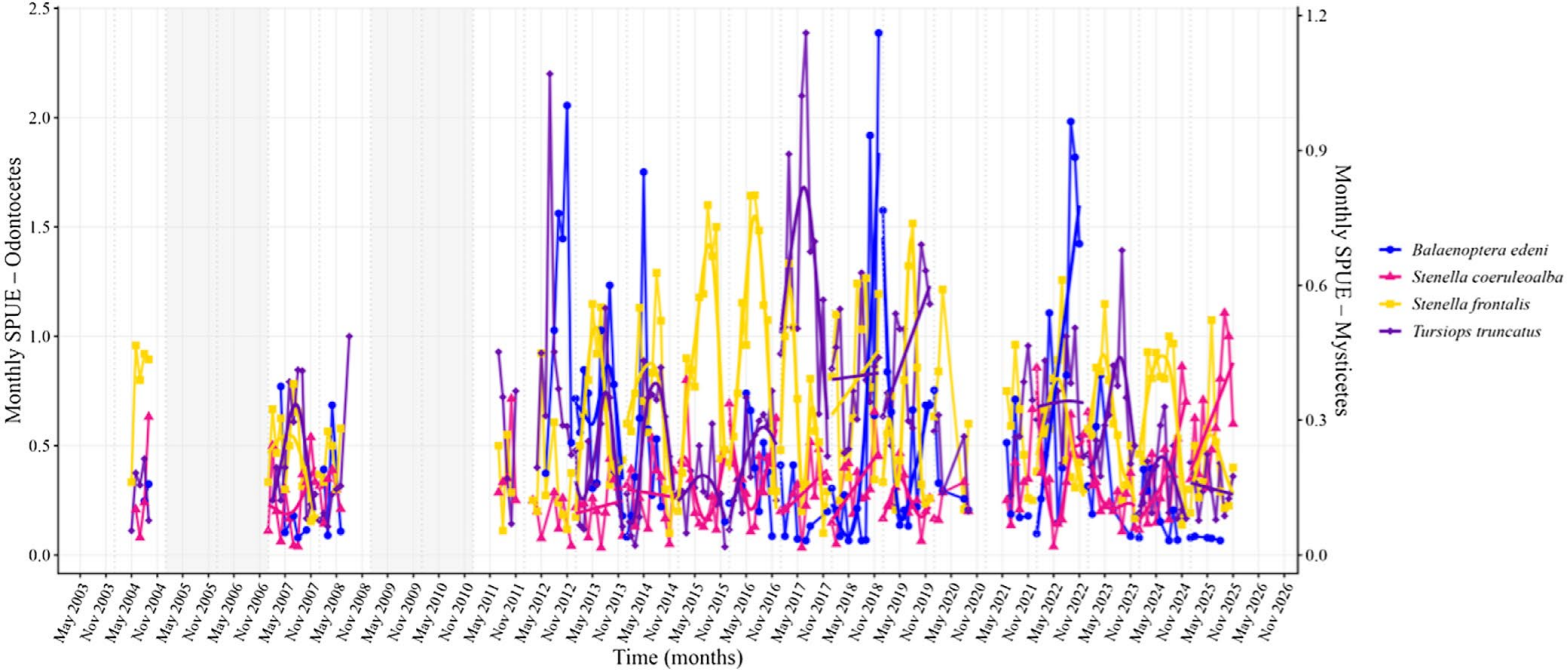
Monthly time series of effort‐standardised cetacean detection rates (SPUE; sightings per surveyed day) for the four most frequently recorded cetacean species in the southwestern waters of Gran Canaria between 2004 and 2025. SPUE values represent species‐specific monthly sightings standardised by survey effort. Periods without survey data (2005, 2006, 2009 and 2010) are shown as gaps in the time series.

The absence of survey data during 2005, 2006, 2009 and 2010 prevented continuous annual comparisons; however, the recurrence of seasonal patterns across multiple years suggests that these temporal signals were not restricted to isolated events (Figure 6).

**FIGURE 7:**
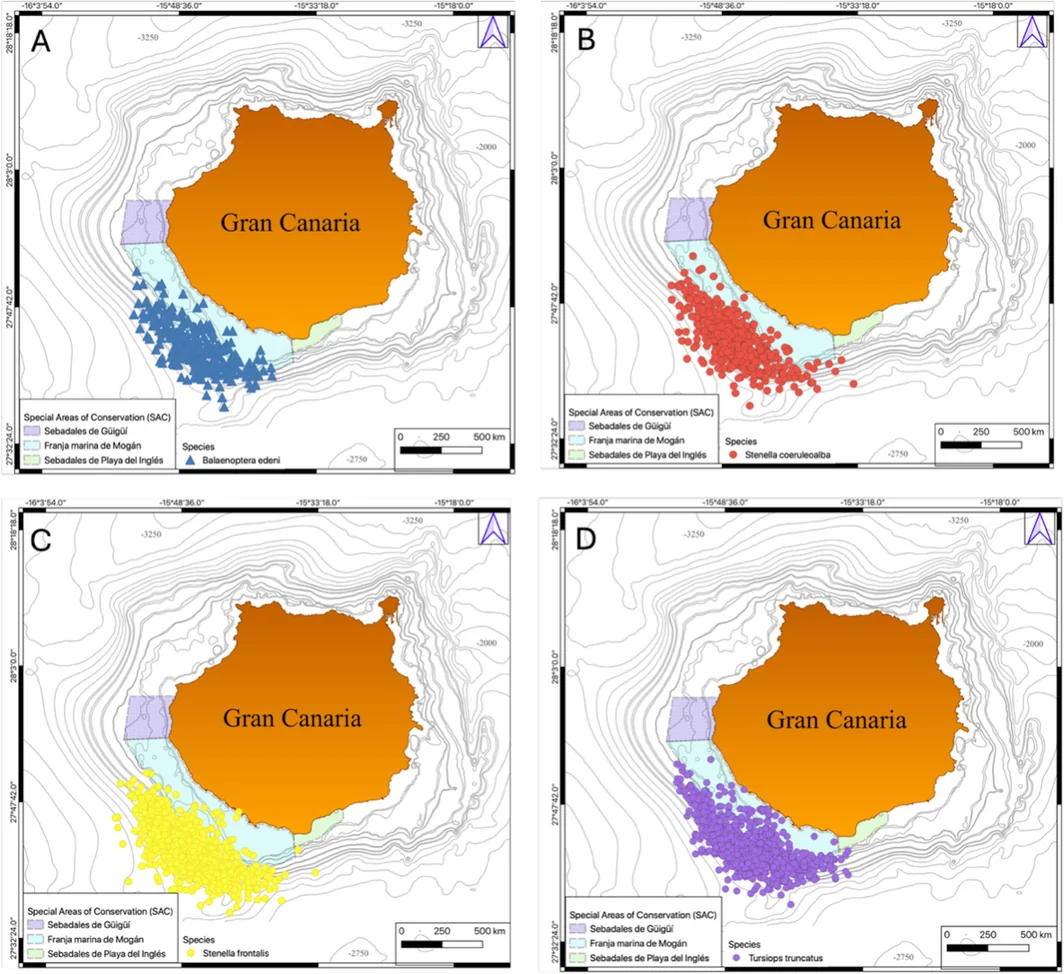
Spatial distribution of recorded cetacean detections from 2004 to 2025 for the four most frequently recorded species in the southwestern waters of Gran Canaria. Maps show detection locations for *Balaenoptera edeni* (A), *Stenella coeruleoalba* (B), *Stenella frontalis* (C) and *Tursiops truncatus* (D), together with the location of Special Areas of Conservation (SACs).

Sightings of *B. edeni* were mainly concentrated along the outer slope of the study area, generally further offshore than those of delphinids. This spatial pattern is consistent with the species' more pelagic ecology and association with deeper waters; however, differences in spatial sampling allocation and species‐specific detectability may also contribute to the observed distribution pattern (Figure 7A).

Sightings of *S. coeruleoalba* were largely concentrated near the edge of the continental shelf but were slightly more inshore than those of *B. edeni* , occurring mainly over intermediate‐depth habitats between oceanic and neritic environments. This distribution is consistent with the broad habitat use described for the species, although differences in detectability and survey coverage may also influence the observed pattern (Figure 7B).

*S. frontalis* detections were predominantly located in coastal and shelf‐associated waters along the southwestern coast of Gran Canaria. This distribution is consistent with the coastal occurrence described for the species in the region, although the concentration of survey effort in coastal areas may also contribute to the observed spatial pattern (Figure 7C).

The most coastal distribution among the four species was observed for *T. truncatus* , with numerous detections near the coast and within areas influenced by local bathymetry. This pattern agrees with the coastal habitat use described for the species in the Canary Islands; however, as for the other taxa, the potential influence of species‐specific detectability and spatial allocation of survey effort should be considered when interpreting these distributions (Figure 7D).

### 3.6 Reproductive Indicators

Calves of *T. truncatus* and *S. frontalis* , the two most frequently sighted odontocetes in the study area, were observed frequently, accounting for 23.6% and 34.0% of all records, respectively, and 57.6% when combined (Table S3). The high frequency of sightings of these species, together with the recurrent presence of mother‐calf pairs and groups of juveniles, suggests that the region functions as an important breeding and rearing habitat for both dolphins. In contrast, *B. edeni* accounted for only 2.9% of total sightings, and records of juveniles were scarce, indicating more occasional use of the area and less consistent reproductive activity for this species of mysticete.

### 3.7 Generalised Additive Model (GAM)

A negative binomial Generalised Additive Model (GAM) was fitted to evaluate temporal and environmental drivers of monthly cetacean sighting counts between 2011 and 2025. Survey effort was incorporated as an offset term to account for differences in sampling intensity among months. The model included species identity as a parametric factor, a general temporal smooth based on the number of months elapsed since January 2011 (*Time_index*), cyclic seasonal variation (*Month*), sea surface temperature (SST) and chlorophyll‐*a* concentration (CHL) as explanatory variables.

The GAM explained 35.3% of the deviance, with an adjusted *R* ^2^ of 0.403 (Table 1). Species identity was highly significant, indicating marked differences in baseline detection levels among taxa. Relative to *Balaenoptera edeni* (reference category), the three odontocete species showed significantly higher expected sighting counts. The strongest positive effect was observed for *Stenella frontalis* (estimate = 1.554 ± 0.097 SE), followed by *Tursiops truncatus* (estimate = 1.458 ± 0.097 SE) and *Stenella coeruleoalba* (estimate = 0.911 ± 0.099 SE) (Table 1).

**TABLE 1:** Results of the negative binomial Generalised Additive Model (GAM) describing monthly cetacean sighting counts between 2011 and 2025.

Parametric coefficients					
Term	Estimate	SE	*z*‐value	*p*	Significance
Intercept	−1.953	0.074	−26.243	< 0.001	***
*Stenella coeruleoalba*	0.911	0.099	9.212	< 0.001	***
*Stenella frontalis*	1.554	0.097	16.063	< 0.001	***
*Tursiops truncatus*	1.458	0.097	15.032	< 0.001	***

Smooth terms					
Smooth term	EDF	Ref.df	Statistic	*p*	Significance
*s*(*Time_index*)	3.163	3.942	17.349	0.002	**
*s*(*Month, cyclic*)	1.228	10.000	2.493	0.112	ns
*s*(*SST*)	1.176	1.328	0.107	0.818	ns
*s*(*CHL*)	1.002	1.005	0.157	0.693	ns

Model statistics	
Statistic	Value
Family	Negative binomial
Link	log
Observations	508
Deviance explained	35.3%
Adjusted *R* ^2^	0.403
Method	REML
REML score	1657.99
*θ*	2.348

*Note:* The model was fitted using monthly sighting counts as the response variable, with survey effort included as an offset term to account for variation in sampling intensity. Parametric coefficients represent species effects relative to the reference category, whereas smooth terms describe nonlinear temporal variation (*Time_index*, months elapsed since January 2011), seasonal variation (cyclic month) and environmental effects (sea surface temperature, SST and chlorophyll‐*a* concentration, CHL). Smooth terms were fitted using penalised regression splines under restricted maximum likelihood (REML). *Time_index* represents the number of months elapsed since January 2011. Significance codes: ***p < 0.001; **p < 0.01; *p < 0.05; *p* < 0.1; ns = not significant.

The temporal smooth component (*s*(*Time_index*)) showed a significant nonlinear pattern throughout the study period (EDF = 3.16, statistic = 17.35, *p* = 0.002), indicating that monthly sighting counts varied over time and did not follow a simple linear trend. The estimated temporal effect showed moderate nonlinear fluctuations across the 2011–2025 monitoring period (Figure S1).

The cyclic seasonal component (*s*(*Month*)) was not significant (EDF = 1.23, statistic = 2.49, *p* = 0.112), indicating limited evidence for consistent intra‐annual variation after accounting for species identity, temporal trends, sampling effort, and environmental predictors. Similarly, environmental predictors showed no significant relationships with monthly sighting counts. Sea surface temperature (SST) was not significant (EDF = 1.18, statistic = 0.11, *p* = 0.818), and chlorophyll‐*a* concentration (CHL) also showed no significant effect (EDF = 1.00, statistic = 0.16, *p* = 0.693). Smooth functions for SST and CHL remained close to zero with broad confidence intervals, suggesting weak and uncertain relationships at the monthly scale (Figure S1).

Model diagnostics indicated adequate model performance and convergence. Residual diagnostics showed no strong systematic deviations between residuals and fitted values, and basis dimension checking did not indicate insufficient flexibility of the smooth terms (Figure S2).

The GAM indicates that temporal variation and species identity were the main contributors explaining variability in cetacean sighting patterns, whereas seasonal and environmental predictors did not provide additional explanatory power at the monthly scale. Descriptive effort‐standardised detection patterns (SPUE) for the four focal species are summarised in Table S4, showing differences in relative detection frequency among taxa. To further characterise species‐specific temporal dynamics, separate negative binomial GAMs were subsequently fitted for *B. edeni* , *S. coeruleoalba* , *S. frontalis* and *T. truncatus* . Results of these species‐specific models, including parametric coefficients, smooth terms and model statistics, are presented in Table S5, while corresponding temporal smooth functions and diagnostic evaluations are shown in Figures S1 and S3–S6.

## 4 Discussion

### 4.1 Ecological Relevance and Comparison With Regional Studies

Our results confirm that the waters off south‐western Gran Canaria support a high diversity of cetacean species, with 20 species recorded over an 18‐year period. This diversity is consistent with previous regional assessments identifying the Canary Islands as an important area for cetacean biodiversity within Macaronesia (Herrera et al. 2021; Martín et al. 2024). The predominance of odontocete detections in our dataset highlights the frequent occurrence of dolphin species in the study area; however, this pattern should be interpreted considering potential differences in detectability among cetacean groups. Coastal distribution, larger group sizes and more conspicuous surface behaviour of many delphinid species may increase their probability of detection from whale‐watching platforms compared with less conspicuous or more offshore species, including several mysticetes.

The Canary Current, subtropical circulation and complex island bathymetry contribute to the development of mesoscale processes, including eddies, filaments and water‐mass retention features, which can enhance productivity and influence prey distribution (Arístegui et al. 2009; Sangrà et al. 2007). These oceanographic processes may contribute to the suitability of south‐western Gran Canaria waters for supporting a diverse cetacean assemblage. However, patterns derived from whale‐watching platforms should be interpreted as variation in effort‐standardised detection rates rather than direct evidence of changes in population size, habitat quality, or ecological status. Species‐specific detectability, spatial allocation of survey effort, behavioural differences and group composition may all influence observed detection patterns.

### 4.2 Temporal Dynamics and the Potential of Long‐Term Monitoring Datasets

The 18‐year time series revealed substantial temporal variability in effort‐standardised cetacean detection rates, extending beyond the expected seasonal component and highlighting the potential of whale‐watching platforms as complementary sources of long‐term ecological information. The increase in SPUE observed between 2013 and 2019 indicates a period of higher relative detection rates, which may reflect a combination of ecological processes (e.g., changes in cetacean distribution, prey availability, or oceanographic variability) and observation‐related factors, including changes in detectability or survey conditions. However, these mechanisms cannot be independently evaluated with the present dataset. Because SPUE represents a relative index of detection patterns rather than absolute abundance, these temporal patterns should not be interpreted as direct evidence of population increases or changes in conservation status.

Although seasonal patterns were evident in effort‐standardised detection rates, the present study does not demonstrate direct ecological changes in cetacean populations. Seasonal variability may reflect a combination of ecological processes, such as species‐specific distribution patterns, prey dynamics and oceanographic variability, but may also be influenced by observation‐related factors. For example, seasonal differences in sea state, weather conditions, spatial distribution of survey effort and species‐specific detectability could affect encounter probability, with surface‐active delphinids occurring in larger groups potentially being easier to detect than less conspicuous offshore mysticetes.

Long‐term datasets derived from non‐systematic platforms can provide valuable information on temporal variability that may be difficult to obtain through short‐term scientific surveys, particularly in regions where continuous dedicated monitoring is limited (González García et al. 2023; Lacetera et al. 2023). By standardising detections according to survey effort, SPUE improves comparability among months and years and allows the identification of broad temporal patterns while partially accounting for variation in sampling intensity. Nevertheless, residual effects of detectability, spatial sampling distribution, group size and species‐specific behaviour may still influence observed trends.

The marked reduction in SPUE during 2020–2021 coincided with the disruption of whale‐watching activity associated with the COVID‐19 pandemic and the consequent reduction in sampling effort. Although detection rates increased afterwards, values remained variable and did not consistently return to pre‐pandemic levels by 2025. This pattern may reflect a combination of factors, including changes in survey coverage, natural variability in cetacean distribution, prey dynamics, or broader environmental fluctuations. Therefore, changes observed during this period should be interpreted cautiously, as they may partly reflect altered observation processes rather than ecological changes. Distinguishing ecological variation from changes in observation processes requires independent datasets and complementary monitoring approaches.

Within the framework of marine monitoring initiatives such as the EU Marine Strategy Framework Directive (MSFD), whale‐watching datasets may provide complementary information for assessing temporal changes in cetacean detection patterns. The combination of long‐term continuity, effort standardisation and species‐specific resolution highlights the potential of SPUE as a complementary metric within broader monitoring frameworks. However, validation against independent abundance estimates, systematic surveys and additional ecological indicators remains necessary before SPUE can be considered a robust operational ecological indicator.

In oceanic archipelagos, where continuous dedicated monitoring programmes can be logistically and economically challenging, integrating data from platforms of opportunity represents a valuable complementary approach for increasing temporal coverage and detecting broad‐scale variability in marine predator occurrence patterns (Piroddi et al. 2022; Thompson et al. 2015). The comparison with temporal patterns reported from neighbouring Macaronesian regions, such as Madeira (Sambolino et al. 2022), provides regional context for interpreting cetacean detection variability. However, because such datasets may share similar methodological limitations, they should not be considered independent validation of the SPUE index. These similarities should therefore be interpreted as evidence of comparable temporal dynamics rather than direct confirmation of ecosystem status or resilience.

### 4.3 Habitat Differentiation, Distribution Patterns and Potential Nursery Function

Seasonal differences among the dominant dolphin species, together with partially overlapping but asynchronous temporal peaks, indicate differences in occurrence patterns throughout the year. These temporal differences may reflect variation in species ecology, behavioural strategies, prey availability, or other factors influencing cetacean presence. Similar differences in seasonal occurrence among sympatric dolphin species have been documented in Macaronesia and other marine ecosystems (Torreblanca et al. 2023).

The frequent occurrence of calves and mother–calf groups in *T. truncatus* and *S. frontalis* suggests that the study area is regularly used by reproductive groups and may provide suitable conditions for early‐life stages. Repeated use of coastal habitats by reproductive groups has been reported in warm, productive island environments (Sambolino et al. 2022). These areas may therefore represent important areas for local cetacean populations and should be considered in management strategies, particularly given potential disturbance associated with maritime traffic and ecotourism activities (Bejder et al. 2006; Martín et al. 2024; Cezimbra et al. 2025).

The contrasting spatial distribution patterns observed between coastal dolphin species and *B. edeni* indicate differences in species occurrence within the study area. The more offshore distribution observed for *B. edeni* , compared with the predominantly coastal occurrence of *S. frontalis* , *S. coeruleoalba* and *T. truncatus* , may reflect differences in habitat association, movement patterns, detectability, or sampling distribution. Similar spatial differences among cetacean species have been reported in relation to environmental gradients and species‐specific behaviour (Parra 2006; Bearzi 2005; Kiszka et al. 2011).

However, because the spatial analyses presented here are descriptive, these patterns should not be interpreted as evidence of habitat partitioning or habitat preference. Rather, they represent differences in observed occurrence and distribution within the surveyed area. Formal spatiotemporal analyses integrating environmental variables, bathymetric features, prey‐related proxies and detection processes would be required to evaluate the ecological mechanisms underlying these patterns.

### 4.4 Environmental Variability and Potential Ecological Mechanisms

Although sea surface temperature (SST) and chlorophyll‐*a* concentration (CHL) are commonly used environmental proxies associated with cetacean distribution through their potential influence on productivity, prey availability and habitat conditions, our analyses provided limited evidence for direct relationships between these surface variables and monthly cetacean sighting patterns. In the general GAM, neither SST nor CHL showed significant effects after accounting for species identity, long‐term temporal variation, seasonal variability and sampling effort. These results indicate that the environmental variables considered here did not explain additional variation in monthly cetacean detections beyond the effects of species‐specific differences and temporal dynamics.

The significant temporal component identified by the GAM indicates that cetacean sighting patterns changed throughout the monitoring period, but these changes were not directly associated with the surface environmental variables included in the model. The absence of significant SST and CHL effects at the monthly scale does not imply that oceanographic conditions are irrelevant for cetacean habitat use. Instead, it suggests that environmental influences may operate through more complex ecological pathways, including indirect effects mediated by prey availability, delayed responses, or interactions among multiple oceanographic processes.

Potential mechanisms linking oceanographic variability and cetacean occurrence include changes in prey distribution, meso‐scale oceanographic features, productivity dynamics and the spatial aggregation of trophic resources. Previous studies in the north‐eastern Atlantic have shown that cetacean distribution can respond to oceanographic variability, although the strength and direction of these relationships are often species‐specific and dependent on the spatial and temporal scale considered (Correia et al. 2021; Pérez‐Jorge et al. 2020).

For *B. edeni* , the lower detection frequency and lower SPUE values observed compared with the three odontocete species suggest a more intermittent occurrence pattern in the study area. This pattern is consistent with a potentially opportunistic use of Canary Island waters, where occurrence may depend on episodic favourable conditions, prey availability, or broader oceanographic processes rather than persistent local surface characteristics (Herrera et al. 2021). Conversely, the consistently high detection frequency of *S. frontalis* , *S. coeruleoalba* and *T. truncatus* indicates a more regular use of the study area, although their temporal variability was not explained by the environmental predictors included here.

Future studies incorporating additional ecological variables, such as prey distribution, primary productivity indices, remotely sensed oceanographic fronts, upwelling indicators and finer‐scale habitat descriptors, may provide further insight into the mechanisms shaping cetacean habitat use in the Canary Islands region (Aguilar de Soto et al. 2020).

### 4.5 Conservation Implications and Management Perspectives

The spatial overlap between areas of high cetacean detection rates and existing Special Areas of Conservation (SACs) provides additional information on the distribution of recurrent cetacean occurrence areas in south‐western Gran Canaria. Although these results do not directly evaluate the effectiveness of current protection measures, identifying areas with repeated cetacean detections can contribute to future management strategies and conservation planning by highlighting locations where monitoring and mitigation measures may be particularly relevant.

The concentration of *T. truncatus* and *S. frontalis* detections in coastal areas, together with the frequent occurrence of calves and mother–calf groups, highlights the need to consider areas of repeated reproductive activity and high cetacean occurrence when developing local management actions. Coastal sectors exposed to maritime activities, including vessel traffic and tourism‐related operations, may require context‐specific approaches aimed at reducing potential disturbance while maintaining sustainable human activities.

Management measures such as voluntary speed reduction, navigation guidelines and recommendations regarding vessel behaviour and density represent practical approaches that may help minimise potential disturbance to cetaceans. Similar interactions between maritime activities and cetacean occurrence patterns have been reported in other areas of the Canary Islands, supporting the need for coordinated regional strategies based on the best available scientific information (Martín et al. 2024).

The whale‐watching sector also represents an important opportunity for conservation‐oriented monitoring. Beyond its socioeconomic relevance, well‐regulated platforms of opportunity can contribute valuable long‐term observations, increase public awareness and complement dedicated scientific surveys. The integration of these datasets with independent monitoring programmes and environmental information may improve future assessments of cetacean distribution patterns, population trends and ecosystem changes (Piroddi et al. 2022).

The results highlight the potential of effort‐standardised whale‐watching observations as a complementary source of information for marine conservation in subtropical archipelagos. However, further validation through independent abundance estimates, systematic surveys and additional ecological indicators is required before SPUE can be incorporated as an operational ecological indicator within formal monitoring frameworks.

### 4.6 Methodological Considerations

This study highlights the value of whale‐watching platforms as cost‐effective contributors to long‐term cetacean monitoring, particularly in regions where sustained dedicated surveys are logistically and economically challenging. However, observations collected from platforms of opportunity are subject to several potential sources of bias, including heterogeneous sampling effort, variable detection probability, weather‐dependent visibility and non‐random survey routes. These limitations should be considered when interpreting temporal patterns and, where possible, addressed through improved effort documentation and complementary monitoring approaches (Neves et al. 2023).

Future monitoring programmes would benefit from integrating visual observations with additional sources of information, including passive acoustic monitoring, automated photo‐identification and AIS‐based effort tracking, to improve estimates of survey coverage, detectability and species‐specific distribution patterns (Thompson et al. 2015; Alves et al. 2013; Almunia et al. 2021; Arranz et al. 2023).

Although SPUE does not provide absolute abundance estimates, it represents a useful effort‐standardised metric of relative cetacean detection patterns in long‐term datasets derived from opportunity platforms. By standardising detections according to survey effort, SPUE improves comparability among periods with different sampling intensity and allows the identification of broad seasonal and interannual patterns. However, this standardisation primarily accounts for variation in sampling intensity and does not fully correct for differences in spatial coverage, species‐specific detectability, or behavioural characteristics that influence encounter probability. Because SPUE is based on the number of detection events rather than the number of individuals observed, temporal variation may also be influenced by changes in group size. In the present dataset, group size showed considerable interannual variability, particularly for *Stenella* species due to occasional large aggregations; however, no consistent temporal increase in group size was observed during periods of higher SPUE.

Species‐specific detectability represents an additional consideration, as cetacean taxa differ in surfacing behaviour, group size, movement patterns and habitat association. For example, the intermittent detection pattern observed for *B. edeni* may partly reflect lower detectability compared with more surface‐active delphinids occurring in larger groups, as well as differences in spatial coverage associated with its more offshore occurrence. Therefore, variation in detections should not necessarily be interpreted as differences in habitat use alone.

Although survey routes followed a consistent operational pattern from the same departure harbour throughout the study period, fine‐scale spatial allocation of effort was not explicitly standardised in the present analysis. Consequently, habitats more frequently accessed during routine whale‐watching operations, particularly coastal areas, may be disproportionately represented, potentially influencing species‐specific detection patterns.

Residual biases related to detectability, environmental conditions, group composition and spatial sampling allocation may therefore influence observed patterns and should be considered when interpreting results. Temporal variation in SPUE should consequently be interpreted as changes in effort‐standardised detection rates rather than direct evidence of changes in population abundance or ecological status. In the absence of independent datasets collected using alternative approaches, such as systematic surveys, passive acoustic monitoring, photo‐identification programmes, or abundance estimation methods, the extent to which observed SPUE trends represent underlying ecological changes cannot be confirmed.

Despite these limitations, long‐term whale‐watching datasets provide valuable complementary information on cetacean temporal variability, particularly in data‐limited regions. Their integration with independent abundance estimates, environmental datasets and standardised monitoring programmes will be essential to evaluate their future applicability within broader ecological assessment frameworks.

## 5 Conclusions

The waters off south‐western Gran Canaria support a diverse cetacean assemblage and represent an important area for long‐term monitoring of cetacean detection patterns in the subtropical Atlantic. The 18‐year dataset revealed substantial seasonal and interannual variability in effort‐standardised cetacean detection rates, with marked differences among species reflecting contrasting temporal sighting patterns, detection frequency and species‐specific ecological traits.

The recurrent detection of mother–calf groups of *T. truncatus* and *S. frontalis* indicates repeated detection of reproductive groups in the study area and highlights the value of long‐term observations for documenting important biological events in the region. Spatial patterns among species further suggest differences in detection distributions, with frequently recorded dolphin species and *B. edeni* showing contrasting associations with coastal and more offshore environments. However, these patterns should be interpreted cautiously because they may be influenced by species‐specific detectability, behaviour and spatial variation in sampling effort.

Our results demonstrate that whale‐watching platforms can provide valuable long‐term information on cetacean detection patterns, particularly in oceanic regions where continuous dedicated monitoring is logistically challenging. Effort‐standardised indices such as SPUE provide a useful descriptive measure of relative detection rates among periods with different sampling intensity and facilitate the identification of broad temporal and seasonal patterns. However, SPUE should not be interpreted as a direct measure of abundance or as a fully validated ecological indicator. The GAM analyses further showed that temporal variation and species identity were the main components explaining variability in cetacean detections, whereas the environmental variables considered here (SST and CHL) did not provide additional explanatory power at the monthly scale.

This study highlights the contribution of platforms of opportunity to long‐term cetacean monitoring in oceanic archipelagos. When combined with complementary ecological, environmental and anthropogenic datasets, whale‐watching observations can provide valuable insights into cetacean detection patterns, temporal variability and conservation priorities. Continued integration with independent monitoring approaches will be essential to validate the applicability of these datasets for assessing broader ecological variability and informing marine conservation strategies.

## Author Contributions

**Inma Herrera:** conceptualization (lead), data curation (lead), formal analysis (lead), investigation (lead), methodology (lead), visualization (lead), writing – original draft (lead), writing – review and editing (lead). **Antonio G. Ramos:** conceptualization (equal), writing – original draft (equal), writing – review and editing (equal). **Ángel Rodríguez‐Santana:** conceptualization (equal), writing – original draft (equal), writing – review and editing (equal). **Ricardo Haroun:** conceptualization (equal), writing – original draft (equal), writing – review and editing (equal).

## Funding

Inma Herrera was supported by a competitive postdoctoral contract awarded by Universidad de Las Palmas de Gran Canaria (PIC‐ULPGC‐2020).

## Conflicts of Interest

The authors declare no conflicts of interest.

## Supporting information


**Table S1:** Number of cetacean sightings per year in southwest Gran Canaria islands waters.

**Table S2:** Monthly number of recorded cetacean sightings in the southwestern waters of Gran Canaria between 2004 and 2025.

**Table S3:** Annual distribution of recorded cetacean calf sightings in the southwestern waters of Gran Canaria between 2018 and 2025.

**Figure S1:** Species‐specific temporal smooth functions estimated from negative binomial Generalised Additive Models (GAMs).
**Figure S2:** Temporal variation in GAM‐predicted cetacean detection rates between 2011 and 2025.
**Figure S3:** Diagnostic plots for the species‐specific GAM of *Balaenoptera edeni* .
**Figure S4:** Diagnostic plots for the species‐specific GAM of *Stenella coeruleoalba* .
**Figure S5:** Diagnostic plots for the species‐specific GAM of *Stenella frontalis* .
**Figure S6:** Diagnostic plots for the species‐specific GAM of *Tursiops truncatus* .
**Table S4:** Effort‐standardised sightings per unit effort (SPUE) for the four focal cetacean species ( *Balaenoptera edeni* , *Stenella coeruleoalba* , *Stenella frontalis* and *Tursiops truncatus* ) between 2011 and 2025.
**Table S5:** Species‐specific Generalised Additive Model (GAM) results.

## Data Availability

The data supporting the findings of this study are available within the article and its Supporting Information. Processed datasets used for the analyses are included as Supporting Information files. Tables S1–, S3 are available as Microsoft Excel (.xlsx) files, whereas Tables S4 and S5 and Figures S1–S6 are included in the accompanying S4 document. Additional information or access to the underlying observational database may be available from the corresponding author upon reasonable request, subject to data ownership and collaboration agreements.
