# Supplementary material for: Long‐Term Fluctuations in Cetacean Occurrence Derived From Whale‐Watching Platforms Off Southwest Gran Canaria: Evaluating Their Potential as an Ecological Indicator
Source: Ecol Evol. 2026 Sep 1;16(9):e74232. doi: 10.1002/ece3.74232 (PMC13531013; doi:10.1002/ece3.74232)
Supplement: Supplementary file 1 [file ECE3-16-e74232-s004.docx]

**Long-term fluctuations in cetacean occurrence derived from whale-watching platforms off southwest Gran Canaria: evaluating their potential as an ecological indicator**

Inma Herrera^1*^, Antonio G. Ramos^1^, Ángel Rodríguez-Santana^2^ and Ricardo Haroun^1^

^1^ Research Group in Biodiversity & Conservation (BIOCON), University Institute ECOAQUA, University of Las Palmas de Gran Canaria (ULPGC), Scientific & Technological Marine Park, Crta. Taliarte s/n, 35214, Telde, Spain

^2^ Oceanografía Física y Geofísica Aplicada (OFYGA), University Institute ECOAQUA, University of Las Palmas de Gran Canaria (ULPGC), 35017, Tafira, Spain

*Corresponding Author**:** Inma Herrera: inma.herrera@ulpgc.es

**Supplementary Material**

**Supplementary Figure S1. Species-specific temporal smooth functions estimated from negative binomial Generalised Additive Models (GAMs).** Estimated smooth effects of temporal variation (Time_index, representing months elapsed since January 2011) on monthly cetacean detection counts for the four focal species (Balaenoptera edeni, Stenella coeruleoalba, Stenella frontalis, and Tursiops truncatus). Solid lines represent fitted smooth functions and shaded areas indicate approximate 95% confidence intervals. Temporal effects were estimated while accounting for variation in sampling effort through an offset term, seasonal variation (cyclic month), species-specific effects, and environmental predictors (sea surface temperature, SST, and chlorophyll-a concentration, CHL).

**Supplementary Figure S2. Temporal variation in GAM-predicted cetacean detection rates between 2011 and 2025.** Monthly predicted detection rates derived from the negative binomial Generalised Additive Model (GAM) for the four focal species. Predictions were obtained from monthly sighting counts while accounting for variation in sampling effort through the offset term. The model included species identity, nonlinear temporal variation (Time_index), seasonal effects (cyclic month), and environmental predictors (sea surface temperature, SST, and chlorophyll-a concentration, CHL).

**Supplementary Figure S3. Diagnostic plots for the species-specific GAM of Balaenoptera edeni.** Diagnostic evaluation of the negative binomial Generalised Additive Model (GAM), including residual patterns, response versus fitted values, quantile–quantile relationships, and basis dimension checking (gam.check). These diagnostics were used to evaluate model convergence, residual behaviour, and the adequacy of smooth-term specification.

**Supplementary Figure S4. Diagnostic plots for the species-specific GAM of** Stenella coeruleoalba**.** Diagnostic evaluation of the negative binomial Generalised Additive Model (GAM), including residual patterns, response versus fitted values, quantile–quantile relationships, and basis dimension checking (gam.check). These diagnostics were used to evaluate model convergence, residual behaviour, and the adequacy of smooth-term specification.

**Supplementary Figure S5. Diagnostic plots for the species-specific GAM of** Stenella frontalis**.** Diagnostic evaluation of the negative binomial Generalised Additive Model (GAM), including residual patterns, response versus fitted values, quantile–quantile relationships, and basis dimension checking (gam.check). These diagnostics were used to evaluate model convergence, residual behaviour, and the adequacy of smooth-term specification.

**Supplementary Figure S6. Diagnostic plots for the species-specific GAM of** Tursiops truncatus**.** Diagnostic evaluation of the negative binomial Generalised Additive Model (GAM), including residual patterns, response versus fitted values, quantile–quantile relationships, and basis dimension checking (gam.check). These diagnostics were used to evaluate model convergence, residual behaviour, and the adequacy of smooth-term specification.

**Supplementary Table S4. Effort-standardised sightings per unit effort (SPUE) for the four focal cetacean species (**Balaenoptera edeni**,** Stenella coeruleoalba**,** Stenella frontalis**, and** Tursiops truncatus**) between 2011 and 2025.** SPUE was calculated as the number of monthly sightings divided by monthly survey effort (survey days) and is presented as a descriptive index of relative detection rates. Detection frequency represents the percentage of monitored months in which at least one sighting of each species was recorded. **Note:** SPUE values are provided for descriptive purposes only and were not used as response variables in the GAM analyses. Generalised Additive Models were fitted using monthly sighting counts as response variables, with survey effort incorporated as an offset term to account for variation in sampling effort.

| **Species** | **Mean SPUE** | **Median SPUE** | **Maximum SPUE** | **Months with detection** | **Total months** | **Detection frequency (%)** |
| --- | --- | --- | --- | --- | --- | --- |
| *Balaenoptera edeni* | 0.150 | 0.0645 | 1.16 | 117 | 184 | 63.6 |
| *Stenella coeruleoalba* | 0.304 | 0.280 | 1.11 | 175 | 184 | 95.1 |
| *Stenella frontalis* | 0.615 | 0.553 | 1.65 | 179 | 184 | 97.3 |
| *Tursiops truncatus* | 0.570 | 0.500 | 2.39 | 179 | 184 | 97.3 |

# **Supplementary Table S5. Species-specific Generalised Additive Model (GAM) results**

Results of species-specific negative binomial Generalised Additive Models (GAMs) describing temporal and environmental variation in monthly cetacean sighting counts for the four focal species (Balaenoptera edeni, Stenella coeruleoalba, Stenella frontalis, and Tursiops truncatus) between 2011 and 2025. Models were fitted using monthly sighting counts as response variables, with survey effort included as an offset term to account for differences in sampling intensity among months. Parametric coefficients represent model intercepts, whereas smooth terms describe nonlinear temporal variation (months elapsed since January 2011), seasonal variation (cyclic month), and environmental effects (sea surface temperature, SST, and chlorophyll-a concentration, CHL).

## **Table S5a. Parametric coefficients**

| **Species** | **Term** | **Estimate** | **SE** | **z-value** | **p-value** | **Significance** |
| --- | --- | --- | --- | --- | --- | --- |
| *Balaenoptera edeni* | Intercept | -2.539 | 0.113 | -22.437 | <0.001 | *** |
| *Stenella coeruleoalba* | Intercept | -1.115 | 0.045 | -24.638 | <0.001 | *** |
| *Stenella frontalis* | Intercept | -0.455 | 0.043 | -10.670 | <0.001 | *** |
| *Tursiops truncatus* | Intercept | -0.575 | 0.034 | -16.694 | <0.001 | *** |

## **Table S5b. Smooth terms**

| **Species** | **Smooth term** | **EDF** | **Ref.df** | **Statistic** | **p-value** | **Significance** |
| --- | --- | --- | --- | --- | --- | --- |
| *B. edeni* | s(Time_index) | 13.610 | 15.977 | 97.821 | <0.001 | *** |
| *B. edeni* | s(Month) | 0.002 | 10.000 | 0.002 | 0.420 | ns |
| *B. edeni* | s(SST) | 1.000 | 1.000 | 0.014 | 0.907 | ns |
| *B. edeni* | s(CHL) | 1.640 | 2.017 | 1.126 | 0.581 | ns |
| *S. coeruleoalba* | s(Time_index) | 3.894 | 4.848 | 18.596 | 0.002 | ** |
| *S. coeruleoalba* | s(Month) | 2.251 | 10.000 | 10.647 | 0.002 | ** |
| *S. coeruleoalba* | s(SST) | 1.000 | 1.001 | 0.140 | 0.709 | ns |
| *S. coeruleoalba* | s(CHL) | 2.465 | 2.964 | 2.678 | 0.386 | ns |
| *S. frontalis* | s(Time_index) | 1.004 | 1.009 | 13.502 | <0.001 | *** |
| *S. frontalis* | s(Month) | 4.247 | 10.000 | 52.840 | <0.001 | *** |
| *S. frontalis* | s(SST) | 1.000 | 1.000 | 0.891 | 0.345 | ns |
| *S. frontalis* | s(CHL) | 1.000 | 1.000 | 0.143 | 0.706 | ns |
| *T. truncatus* | s(Time_index) | 12.405 | 14.818 | 165.516 | <0.001 | *** |
| *T. truncatus* | s(Month) | 2.587 | 10.000 | 17.359 | <0.001 | *** |
| *T. truncatus* | s(SST) | 1.000 | 1.001 | 0.105 | 0.746 | ns |
| *T. truncatus* | s(CHL) | 2.052 | 2.509 | 1.117 | 0.517 | ns |

## **Table S5c. Model statistics**

| **Species** | **Family** | **Link** | **Observations** | **Deviance explained (%)** | **Adjusted R²** | **Method** | **REML score** | **θ** |
| --- | --- | --- | --- | --- | --- | --- | --- | --- |
| *Balaenoptera edeni* | Negative binomial | log | 127 | 55.9 | 0.464 | REML | 285.059 | 1.442 |
| *Stenella coeruleoalba* | Negative binomial | log | 127 | 29.3 | 0.432 | REML | 372.495 | 7.287 |
| *Stenella frontalis* | Negative binomial | log | 127 | 37.4 | 0.442 | REML | 445.514 | 6.079 |
| *Tursiops truncatus* | Negative binomial | log | 127 | 66.0 | 0.650 | REML | 424.892 | 13.686 |

**Note:** Time_index represents the number of months elapsed since January 2011. Smooth terms were fitted using penalised regression splines under restricted maximum likelihood (REML). EDF indicates the estimated degrees of freedom of each smooth term. Significance codes: *** p < 0.001; ** p < 0.01; * p < 0.05; . p < 0.1; ns = not significant.
